# Investigating the Role of Indoleamine 2,3-Dioxygenase in Acute Myeloid Leukemia: A Systematic Review

**DOI:** 10.3389/fimmu.2021.651687

**Published:** 2021-03-10

**Authors:** Georgia Wells, Paul T. Kennedy, Lekh N. Dahal

**Affiliations:** Department of Pharmacology and Therapeutics, Faculty of Life and Health Sciences, Institute of Systems, Molecular and Integrative Biology, University of Liverpool, Liverpool, United Kingdom

**Keywords:** acute myeliod leukemia, regulatory T cells, indoleamine 2,3-dioxygenase, prognosis, 1-Methyltryptophan

## Abstract

**Background:** The immunomodulatory enzyme, indoleamine 2,3-dioxygenase (IDO) facilitates tryptophan catabolism at the rate-limiting step of the kynurenine (Kyn) pathway. IDO expression and elevations in Kyn metabolites are associated with immunosuppressive tumor microenvironment including T cell proliferative arrest and generation of regulatory T cells (Tregs) which can favor tumor progression. However, the extent of the role of IDO in acute myeloid leukemia (AML) is currently ill-defined. This study reviews the role of IDO-driven Treg function in AML and evaluates the current body of evidence implicating IDO in AML pathogenesis.

**Method:** Studies related to IDO in AML were identified through a systematic review of PubMed and Scopus. Data extracted described sample analysis, IDO expression, IDO in prognosis, techniques used in Treg phenotypic studies, and the effect of IDO inhibitors.

**Results:** Twenty studies were included in the systematic review. Expression of IDO was identified in a range of cells in AML, both inducible and constitutive. Seven studies indicated an association between elevated expression and poor clinical prognosis. Six studies suggested a positive correlation between IDO expression and Treg induction, with FoxP3 being the prominent Treg phenotypic marker. Of eight studies investigating IDO inhibition, some reported reductions in Treg frequency and enhanced effector T cell proliferation.

**Conclusion:** This review highlights that IDO expression in AML is associated with poor prognosis and measurement of IDO and its Kyn metabolites may offer utility as prospective prognostic markers. Pharmacological inhibition of IDO using novel drugs may hold promise for the treatment of AML.

## Introduction

Acute myeloid leukemia (AML) is a hematological malignancy which interferes with the normal haematopoietic production of blood cells. It is characterized by the accumulation and expansion of immature myeloid cells (blasts) within the bone marrow that proliferate rapidly and inhibit differentiation of the normal progenitor cells (1). AML is primarily a disease of adulthood and comprises ~80% of hematological malignancies observed in patients over 60 (2, 3). AML is typically treated by a standard induction chemotherapy of a 7 + 3 regimen of cytarabine with an anthracycline (daunorubicin or idarubicin), genetic risk profiles then determine follow up treatment of consolidation chemotherapy or allogeneic hematopoietic stem cell transplantation (4). The efficacy of standard induction chemotherapy is limited and is followed by AML relapse and mortality in a majority of patients over 60 (4, 5).

The “two-hit” model of AML leukaemogenesis proposes that two mutation classes drive the disease, those that augment proliferation and cell survival (e.g., FLT3) and those which disrupt haemopoietic differentiation and apoptosis (e.g., CBFβ-MYH11) (6). Recent development of therapies targeting such mutations including the FLT3 inhibitors, lestaurtinib, and midostaurin have improved patient outlook (7). These have been shown to produce clinical responses, but they are often transient and subject to resistance mechanisms (8). Overall, current standard of care regarding chemotherapeutics is limited, with prognosis remaining poor for many patients, warranting the need for detailed biological understanding of disease pathogenesis to develop future therapies (9). A deeper understanding of the molecular mechanisms underlying AML pathogenesis may offer new avenues to successful targeted therapies.

The heterogeneity of immune response between patients has made it difficult to characterize the landscape of AML tumor microenvironment. Several components within the tumor microenvironment interact to reprogram tumor initiation, progression, and response to therapies. Malignancies like AML deploy a plethora of immune escape mechanisms, including evasion of immunosurveillance systems, whereby cancer cells are able to avoid destruction (10). Such escape mechanisms in AML include aberrant expression of immune checkpoint receptors, deregulation of tumor necrosis receptor families and ligands which regulate T cell and natural killer cell responses, and immunomodulatory enzymes which abrogate anti-tumoral T cell effector function (11, 12).

Indoleamine 2,3-dioxygenase (IDO) is a haem-containing, immunomodulatory enzyme that catalyzes the oxidative cleavage of the indole ring of the essential amino acid tryptophan (Trp), producing immunoregulatory metabolites, collectively known as kynurenines [Kyn(s)] (13). IDO was originally described to promote immune tolerance in mammalian pregnancy, chronic infection, autoimmunity and allergic inflammation, but it has now become evident that it plays an important role in suppressing antitumour responses, and promoting tumor resistance (14). Some of the Kyn metabolites of tryptophan catabolism are known to inhibit T cell proliferation by arresting the cell cycle and inducing apoptosis (15). Direct action of Kyn has been associated with the generation of regulatory T cells (Tregs) (15). Natural Tregs, characterized by their constitutive expression of surface markers, such as CD25, CTLA-4, and the forkhead box P3 (FoxP3) transcription factor, are able to aid evasion of immune surveillance and suppress cytotoxic CD8^+^ T cell proliferation, favoring tumor progression (16).

Defining the tumor microenvironment and immune escape mechanisms of AML will support the development of new therapeutic strategies. IDO is of particular interest in this regard as studies report IDO expression in both bone marrow and in peripheral blood AML blasts, but the role it plays in disease progression remains unclear (12). Research aimed at defining tumor resistance and identifying immunosuppressive mechanisms in AML is expanding, but there is a lack of consolidated review that aims to systematically decipher the role of this immunosuppressive enzyme. Therefore, this study will review the role of IDO-driven Treg function in AML and evaluate the current body of evidence implicating IDO in AML pathogenesis, to determine its indication in prognosis and as a potential therapeutic opportunity.

## Methods

A systematic review of literature was performed to identify the role of IDO AML progression. The review was conducted according to the guidelines outlined in the PRISMA statement (Supplementary Table 1) (17).

### Search Strategy

A systematic search of PubMed (performed in October 2020) was conducted to identify appropriate studies. The following keyword combinations were used:

1. “Indoleamine 2,3-dioxygenase” and “AML”
2. “Indoleamine 2,3-dioxygenase” and “Acute Myeloid Leukemia”
3. “1-Methyltryptophan” and “Acute Myeloid Leukemia”
4. “1-Methyltryptophan” and “AML”
5. “1-MT” and “Acute Myeloid Leukemia”
6. “1-MT” and “AML”
7. “IDO” and “Acute Myeloid Leukemia”
8. “Tryptophan” and “Acute Myeloid Leukemia”

An additional search was conducted in the Scopus database using the keywords “Indoleamine 2,3-dioxygenase” and “AML”. Reference lists of identified papers were also scrutinized to identify additional sources.

Inclusion criteria for the systematic review were: Studies based on human cells and/or cell lines; Studies that assessed the role of IDO in AML; Studies published in English.

Exclusion criteria for the review were: Review/Systematic review articles, case studies, letters to the editors, books, and abstracts; Studies focusing on other cancer types, such as chronic myeloid leukemia (CML), acute lymphocytic leukemia (ALL) etc.; Studies primarily based on animal models of AML and AML studies that did not investigate IDO.

### Data Extraction

Studies satisfying the inclusion criteria were independently assessed and a data extraction was conducted to obtain the following data: Authors, journal, publication year, sample size, sample type, sample analysis method, IDO expression, role of IDO in prognosis, Treg markers identified, role of Tregs in AML, and the effect of IDO inhibitors. Due to the heterogenous nature of findings and variations in study designs, number of samples and methods employed for quantitation of IDO, meta-analysis of the results was not feasible.

## Results

**Figure 1:**
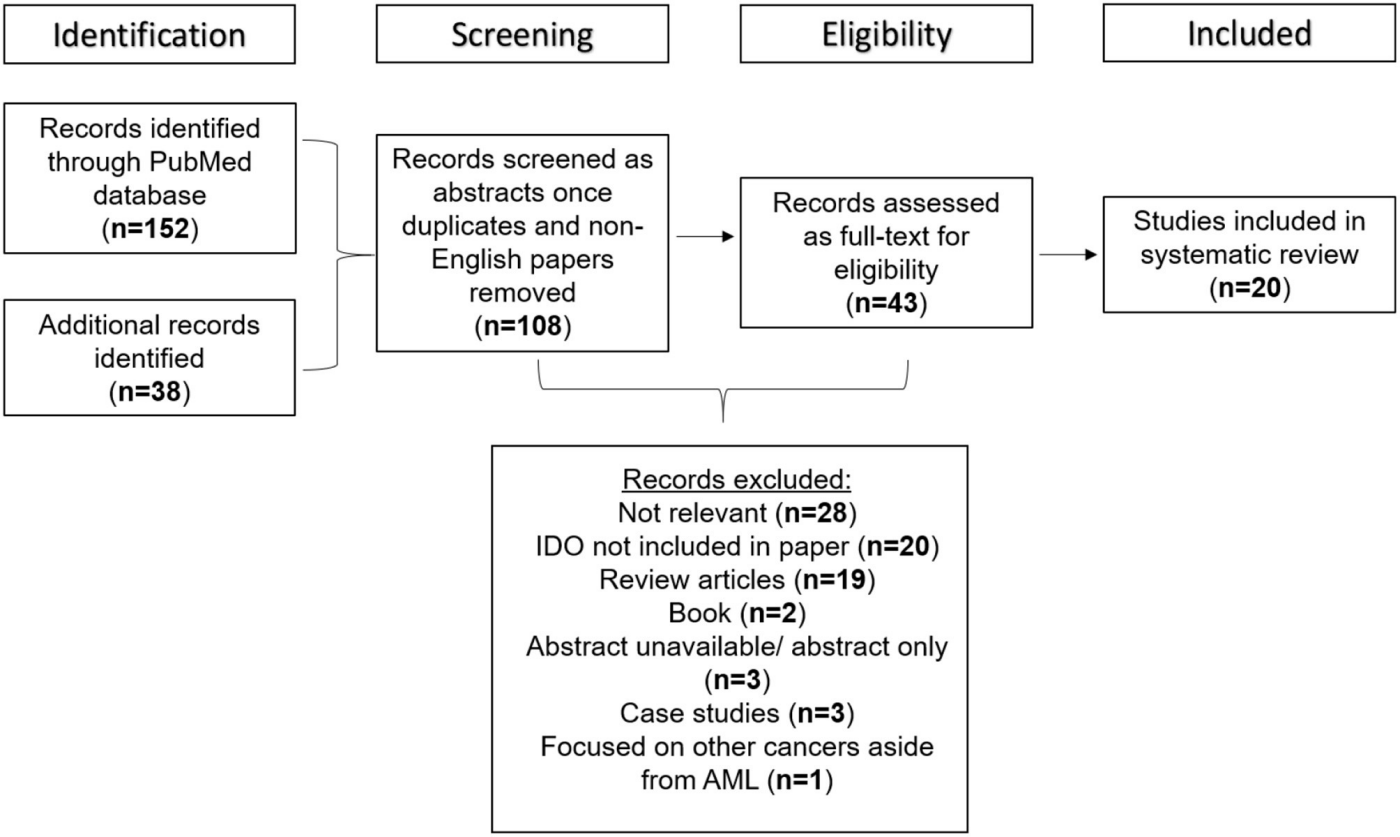
Flow diagram detailing the studies retrieved for the systematic review.

Of 190 papers identified through PubMed and Scopus search, 108 met the inclusion criteria once duplicates and non-English papers were removed. When full texts were screened, a final 20 studies were found to be suitable for inclusion in the study. The reasons for manuscript exclusions were those that had no mention of both IDO and AML (*n* = 28), manuscripts that did not tackle the role of IDO in AML (*n* = 20), those with unavailable abstracts or only abstracts were available (*n* = 20), review articles (*n* = 19), books (*n* = 2), those based on case studies alone (*n* = 3), and those focused on other types of cancer aside from AML (*n* = 1). A flow diagram detailing the screening process used to determine the studies suitable for inclusion in the systematic review is shown in Figure 1.

### Data Summary

The basic characteristics of the studies included in this review are summarized in Table 1.

**Table 1:** Summary of the basic characteristics of the acute myeloid leukemia (AML) study samples included in the systematic review.

**Study**	**Type of sample**	**Sample number**
Arandi et al. (12)	Peripheral blood	37 *de novo* Cytogenetically normal-AML 22 Healthy controls
Mansour et al. (18)	Bone marrow	12 *de novo* adult AML patients 8 Healthy controls
Folgiero et al. (19)	Peripheral blood Bone marrow	37 child AML patients
Fukuno et al. (20)	Bone marrow	62 *de novo* adult AML patients
Corm et al. (21)	Blast cells Sera	184 adult AML patients
Mangaonkar et al. (22)	Bone marrow	40 adult AML patients
EL Kholy et al. (23)	Peripheral blood	25 adult AML patients 25 Healthy controls
Curti et al. (24)	Bone marrow Peripheral blood	76 AML patients
Mabuchi et al. (25)	Bone marrow	48 adult AML patients
Curti et al. (26)	Peripheral blood	8 AML patients
Hara et al. (27)	Bone marrow	29 adult AML patients
Martine et al. (28)	Bone marrow Peripheral blood	356 AML patients
Wang et al. (29)	Bone marrow Peripheral blood Serum	55 *de novo* AML patients 45 Healthy controls
Chen et al. (30)	Bone marrow Peripheral blood	49 AML patients 11 ALL patients 15 Healthy controls
Curti et al. (31)	Bone marrow Primary AML cells	76 AML patients
Trabanelli et al. (32)	Peripheral blood Buffy coats	Unspecified number of AML patients and healthy controls
Folgiero et al. (33)	Bone marrow Buffy coats	Unspecified number of child AML patients and healthy controls
Zhou et al. (34)	Heparinized blood samples	102 AML patients 102 Healthy controls
Lecciso et al. (35)	Bone marrow Peripheral blood	23 AML patients Unspecified number of healthy controls
Iachininoto et al. (36)	HL-60 cells	–

A substantial majority of the studies selected focused on human samples alone (12, 18–23, 25–34). One study assessed human samples and incorporated some aspects of murine AML biology (24), one study looked at human samples, a murine model and HL-60 cell lines (35) whilst one exclusively HL-60 cells (36). Of the studies focusing on human samples, 10 were based on samples taken from adults (≥18 years) (12, 18, 20–23, 25, 27, 31, 35), one from child samples (19), whilst eight did not specify subject age (24, 26, 28–30, 32–34). Some of the studies used AML classification systems; 11 studies applied the French-American-British (FAB) classification (12, 19, 20, 23, 25, 27–31, 35) and five used cytogenic risk categories (22, 27–29, 31). In eight of the studies AML type was categorized as either *de novo* AML (12, 18, 20, 25, 27, 29), cytogenetically normal AML (12) and AML where complete remission (CR) has been achieved (32).

In relation to the methodologies employed by the studies for the quantitation of IDO, seven studies focused directly on IDO1 (19, 22, 26, 32, 33, 35, 36), whilst the remaining studies were non-specific in the type of IDO. None of the studies focused on IDO2 alone. The most common method, used in 15 of the 20 studies, to detect IDO mRNA expression was polymerase-chain reaction (12, 19–24, 26–28, 30–32, 34, 36). IDO protein expression was detected using western blotting in seven studies (21, 26, 28, 31, 33, 35, 36), immunohistochemistry in three studies (18, 22, 30), and immunocytochemistry in two studies (24, 31). Nine studies focused on IDO activity by measuring Trp and Kyn levels using high-performance liquid chromatography (HPLC) (19, 21, 24–27, 29, 33, 36). Some additional methods used in the studies included the measurement of IDO activity by colorimetric assay (Bradford method) (23), spectrophotometric analysis (32), and fluorescent detection (34).

Of the 20 studies identified, eight investigated the role of Tregs in relation to IDO (12, 18, 19, 24, 26, 32, 35, 36). The main methodology used was flow cytometry in five of the studies (18, 19, 32, 35, 36), with T cell surface staining (19, 36), standard mixed lymphocyte reaction (24, 26), polymerase-chain reaction (12), phenotypic and functional assays (26), and mixed tumor lymphocyte cultures (19, 36).

### Role of IDO in AML

Methodologies used and the proposed role of IDO within AML are summarized in Table 2.

**Table 2:** Summary of the methods for detection of indoleamine 2,3-dioxygenase (IDO) and regulatory T cell (Treg) markers, and the suggested role of IDO and Tregs in AML progression and prognosis.

**Study**	**Method for detecting IDO**	**Suggested role of IDO in AML**	**Method for detecting Tregs**	**Treg markers**	**Association between Tregs and IDO**
Arandi et al. (12)	Real-time PCR	High IDO expression in peripheral blood mononuclear cells is associated with the promotion of disease progression, poor immune response, and poor prognosis.	Real-time PCR	FoxP3	FoxP3 expression is upregulated in AML patients. Positive correlation observed between IDO and FoxP3 expression
Mansour et al. (18)	Immunohistochemistry	IDO expression in mesenchymal stem cells has a positive correlation with bone marrow blasts. Modulating the immune system may improve patient survival in AML.	Flow cytometry	CD4 CD25	IDO expression in mesenchymal stem cells positively correlated with percentage of Tregs
Folgiero et al. (19)	Real-time quantitative PCR, reverse phase HPLC	IDO protein and activity is not constitutively expressed. Ability to increase IDO expression is exclusively assigned to FAB-M4/M5 subtypes.	T cell staining, Flow cytometry	FoxP3	IDO-expressing AML blasts favor the emergence of FoxP3-expressing Tregs
Fukuno et al. (20)	Real-time reverse transcription PCR	IDO expression is not significantly different between FAB subtypes and cytogenic risk profiles but is associated with poor prognosis.	–	–	–
Corm et al. (21)	Real-time quantitative PCR, WB, HPLC	There is no correlation between IDO mRNA and Fab subtypes. IDO may be expressed constitutively in come patients, but blast stimulation may be required in others.			
Magaonkar et al. (22)	Real-time quantitative PCR, Immunohistochemistry, HPLC	IDO mRNA has a negative correlation with overall survival and high levels IDO found to be an independent predictor of poor outcomes.	–	–	–
El Kholy et al. (23)	Colorimetric assay, reverse transcriptase PCR	Mononuclear cells expressing IDO mRNA also have functional activity. Increased IDO activity is a negative prognostic sign in AML.	–	–	–
Curti et al. (24)	Real-time PCR, Immunocytochemistry, HPLC	IDO can be constitutively expressed. Production of IDO in AML cells directly increases the number of Treg cells.	Flow cytometry, SMR	CD4 CD25	Treg cells in IDO-expressing AML are induced by the conversion of CD4^+^CD25^−^ T cells into CD25^+^ T cells
Mabuchi et al. (25)	HPLC	High serum concentrations of Kyn are associated with poor outcomes.	–	–	–
Curti et al. (26)	Real-time quantitative PCR, WB, reverse phase HPLC	IDO is expressed in both immature and mature dendritic cells, but marked upregulation is seen in the later stages of dendritic cell generation.	SMR, phenotypic and functional assays	CD4 CD25	AML-like dendritic cells have increased percentage Tregs which reduce T cell proliferation in culture
Hara et al. (27)	PCR, HPLC	Combination of elevated Kyn levels and IDO mRNA are associated with poor outcomes in AML.	–	–	–
Martine et al. (28)	Real-time quantitative PCR, WB	No difference in IDO mRNA between FAB subtypes and cytogenic risk profiles. High IDO expression is associated with poor outcomes.	–	–	–
Wang et al. (29)	UHPLC-Q-TOFMS	AML patients exhibit higher levels of Kyn, suggesting IDO expression increases cancer burden.			
Chen et al. (30)	Immunohistochemistry, Real-time fluorescence PCR	IDO mRNA expression is highest in AML-M5 subtypes and is associated with poor prognosis.	–	–	–
Curti et al. (31)	Immunocytochemistry, WB, Reverse transcriptase PCR	AML samples can show constitutive expression of IDO. AML-M5 has higher, but non-significant, expression in IDO positive cells.	–	–	
Trabanelli et al. (32)	Real-time quantitative PCR, Spectrophotometric analysis	Presence of PGE2 can upregulate IDO mRNA in dendritic cells suggesting modulation role in the expression and function of IDO.	T cell staining, FC	CD4 CD25 FoxP3	Addition of an IDO inhibitor reduced Tregs suggesting a IDO1 mediated induction of Tregs in dendritic cells, in the presence of PGE2
Folgiero et al. (33)	Reverse phase HPLC, WB	IDO positive AML blasts can down-regulate natural killer cell degranulation, thus favoring immune escape.	–	–	–
Zhou et al. (34)	Real-time PCR, fluorescence detection	Redox protein, thioredoxin, correlates with IDO and could contribute to aggressive tumor growth.	–	–	–
Lecciso et al. (35)	WB	ATP release from daunorubicin chemotherapy treatment may promote the IDO pathway.	FC	CD4 CD25 FoxP3	ATP-mediated upregulation of IDO1 in mature dendritic cells correlated with the generation of Tregs
Iachininoto et al. (36)	Real-time quantitative PCR, Reverse phase HPLC	Cox-2 pathway may regulate IDO expression in AML cells. Inhibition of the Cox-2/PGE2 pathway may constrain the leukemia-induced immune response.	T cell staining, FC	FoxP3	Conversion of CD4^+^ T cells to *bona fide* Tregs promoted in IFN-γ challenged HL-60 cells

*PCR, polymerase-chain reaction; HPLC, high-performance liquid chromatography; WB, western blot; SMR, standard mixed lymphocyte reaction; UHPLC-Q-TOFMS, ultra-high-performance liquid chromatography-quadrupole time-of-flight mass spectrometry*.

IDO expression was identified in peripheral blood mononuclear cells (12, 23, 31), but not in bone marrow-derived mononuclear cells (31). Its expression was also identified in bone marrow-derived mesenchymal stem cells (18) and dendritic cells (DCs) (21, 25, 26). Four of the 20 studies reported elevated IDO expression in AML patients compared to healthy controls (12, 18, 23, 30). One study identified elevated Kyn levels in AML patients compared to healthy controls (29).

Three studies identified constitutive IDO expression by leukemic cells of some AML patients (21, 24, 31). One study found no constitutive IDO expression on leukemic blasts from peripheral blood and bone marrow but identified induction by IFN-γ (19). This was supported by two studies indicating the ability of IFN-γ to induce IDO at the molecular level in samples which lacked constitutive expression of IDO (21, 31).

Correlations between IDO expression and FAB types were investigated in seven of the studies reviewed (19–21, 25, 28, 30, 31). Four suggested no significant difference in IDO expression or Kyn levels between subtypes (20, 21, 25, 28). Of the remaining studies, one identified higher IDO expression in the FAB-M5 subgroup compared to non-M5 and healthy controls (1.3- and 3.1-fold, respectively) (30), one found upregulated IDO expression in FAB-M4/M5 subgroups compared to no upregulation in FAB-M1/M2 groups (19). One final study identified higher IDO-positive expression in the FAB-M5 subgroup, although this was not statistically significant (31).

Three studies assessed correlations between cytogenic risk profile and IDO expression, with all concluding that IDO expression has no correlation with cytogenic risk profile (19, 20, 28). Additional factors influencing the IDO pathway were assessed in five of the studies (32–36). Two studies identified the key role of the cyclooxygenase-2/Prostaglandin E2 (COX-2/PGE2) pathway in the upregulation of IDO expression and function in AML cells (32, 36), suggesting modulation of IDO can occur as a result of the environmental cytokine composition (32). These studies suggest that inhibition of the COX-2/PGE2 pathway (36) and DC based vaccines (32) may constrain leukemia-induced immune suppression.

Seven studies investigated the correlation between IDO expression and the percentage of blasts in either bone marrow or peripheral blood (12, 18, 20, 23, 25, 27, 31). One study identified a positive correlation between IDO expression and bone marrow blast cell percentage in bone-marrow derived mesenchymal cells (18), whilst four studies found no significant correlation between IDO expression and blast percentage in bone marrow (12, 20, 23, 27), and one established no correlation between IDO expression and mean percentage of peripheral blood blasts (12). Two further studies found no significant correlation between Kyn levels and peripheral blood and bone marrow blast percentage (25) and comparable circulating blast cell counts between IDO-positive and IDO-negative patients (31).

One study identified that IDO positive expression on AML blasts reduces natural killer cell degranulation, thereby promoting immune escape (33). Another study investigated the involvement of oxidative stress in relation to IDO expression, finding a positive correlation between IDO and the redox protein, thioredoxin (34). The final study found that ATP release from chemotherapy treatments (especially daunorubicin) can promote the IDO pathway, suggesting that current chemotherapy treatments may drive the immune suppressive environment in AML *via* IDO upregulation (35).

### IDO and Prognosis

Regarding prognosis, seven studies associated high IDO expression/activity (and its related elevated Kyn levels) with poor prognosis (12, 20, 23, 25, 27, 28, 30). This poor outcome was linked specifically to IDO mRNA expression in two studies (27, 30). In terms of survival, overall survival (OS) was reduced in IDO-positive groups compared to IDO-negative groups in three studies (19, 20, 27) (Table 3). However, one paper did not correlate IDO mRNA with OS (21).

**Table 3:** Overall survival data (%) for IDO-positive and IDO-negative AML samples.

**Study**	**Years**	**Overall survival of patients (%)**	
		**IDO-positive**	**IDO-negative**
Folgiero et al. (19)	8	31.6	63
Fukuno et al. (20)	3	39	74
Hara at el. (27)	3	37	77

Fewer IDO-positive patients achieved CR than IDO-negative patients in three of the studies reviewed (20, 27, 28). One study found that CR was similar between the IDO-positive and -negative groups, but did identify relapse cases consisted of mainly IDO-positive patients (19). CR was lower in patients with high Kyn levels in one study (27), but there was no significant difference in CR based on Kyn levels in another (25). However, 3-year OS associated with these studies showed that high Kyn levels (≥2.4 μM) resulted in lower overall OS (25, 27).

Five of the studies investigating the effect of IDO and its consequent elevation of Kyn levels also identified key prognostic markers for patient outcomes (20, 25, 27–29). One study identified IDO expression as a useful prognostic factor (28) and a further three studies specifically identified IDO mRNA as a potential candidate (20, 27, 29). However, one suggested that IDO mRNA could be used independently from existing molecular markers (20), whilst another suggested this marker is not independent of other factors (29). Measurement of Kyn levels were also identified as a useful prognostic factor in two studies (25, 27), with one of the studies indicating that the prognostic value of both IDO mRNA and measure of Kyn levels in combination can be associated with poor outcomes (27).

### Relationship Between IDO Expression and Treg Abundance

The link between IDO expression and Treg abundance was addressed in eight studies (12, 18, 19, 24, 26, 32, 35, 36). The methods, markers and roles used are summarized in Table 2. FoxP3 was the predominant Treg phenotypic marker, used as a sole marker in three studies (12, 19, 36), or in combination with CD4 and CD25 in two studies (32, 35). The remaining studies identified Tregs through the expression of the markers CD4 and CD25 (18, 24, 26). One study identified Treg subpopulations using a combination of the following markers: CD3/CD4/CD25/CD127/CD15s/CD45A/FoxP3/PD-1/Ki67 (35).

An increase in Treg abundance in AML patients compared to control groups was identified in three studies (12, 18, 24), whilst six studies identified a positive correlation between IDO expression and Treg percentage (12, 18, 19, 24, 26, 35). This correlation was shown in mesenchymal stem cells (18), IFN-γ activated AML blasts (19), and AML-like DCs (26). Generation of Tregs was also suggested in DCs *via* an IDO mediated mechanism in the presence of PGE2 (32). One study showed that the CD4^+^CD25^+^ Treg phenotype significantly reduced T cell proliferation in culture (26), and another suggested IDO elevation may be associated with an increased Treg frequency (12). One study also found that tumor microenvironment enriched with IDO-expressing AML cells could convert CD4^+^CD25^−^ T cells into CD4^+^CD25^+^ T cells *via* tryptophan catabolism (24).

### Effect of IDO Inhibition

Of the 20 studies, eight included experiments using an IDO inhibitor (21, 23, 24, 26, 31, 32, 35, 36), whilst three studies suggested IDO inhibition could be used therapeutically in AML treatment (22, 27, 28). Six of these studies included the IDO inhibitor 1-methyl-tryptophan (1-MT) (23, 24, 26, 31, 32, 35). 1-MT reduced Treg frequency (24, 32, 35) and Kyn production in IDO-expressing DCs (26) and increased T cell proliferation in IDO-positive cells but not IDO-negative cells (31). One study explored the effect of 1-MT in combination with the chemotherapeutic agent Adriamycin, establishing that the combination was significantly more effective in reducing blast cell proliferation than Adriamycin alone (23).

Other IDO inhibitors used included pyrrolidine dithiocarbamate, abrogated Kyn production (21) and the cyclooxygenase-2 inhibitor, Nimesulide, which abrogated Kyn release and inhibited Treg differentiation to a lesser extent than 1-MT (36). Overall, two of the studies suggested IDO inhibition in adjuvant to chemotherapy may be a means to clear residual leukemic cells and mount an effective anti-tumoral immune response (21, 23).

## Discussion

IDO has been shown to play an important role in the maintenance of immune tolerance, whilst also being implicated in several autoimmune inflammatory diseases, chronic infections and cancers (37). Its activity is majorly attributed to the enzymatic catabolism of the essential amino acid Trp, producing metabolites which arrest T cell proliferation, induce T cell apoptosis, and generate Tregs, producing an immune suppressive environment able to evade immunosurveillance systems. In AML, there is little biological understanding of disease pathogenesis resulting in high mortality rates despite efforts to refine current techniques and the development of targeted therapies. IDO and its Treg-inducing environment are not well-characterized in AML and so the clinical outlook for patients expressing IDO remains unclear.

This systematic review evaluates current knowledge on the role of IDO in AML, aiming to decipher its contribution to disease progression, prognosis, and potential therapeutic interventions. It is clear from the majority of the studies that IDO can be expressed on mononuclear cells of the peripheral blood, bone-marrow derived mesenchymal cells as well as leukemic blasts themselves (12, 18, 19, 21, 23–26, 31). Methodologies across these studies were consistent, focusing of peripheral blood and bone marrow, and utilizing PCR techniques. Only mesenchymal cell IDO expression was identified using bone marrow-derived samples with immunohistochemistry. This consistency across methodologies and samples used suggests that the expression of IDO is variable across multiple cell types. However, there occurs to be some discrepancy whether IDO is constitutively expressed (21, 24, 31) or induced (19, 21, 31). Consensus stands that whilst a subset of patients may have constitutive expression of IDO, others require it to be induced. These findings indicate an inter-patient variability in IDO expression in AML and that further studies should assess expression on a case-by-case basis to overcome the discrepancy. It also suggests that constitutive expression of IDO is not necessary to the progression of AML, but that it may favor progression.

In this review, a controversy was also highlighted regarding IDO expression relating to FAB subtypes. Some of the studies suggested that higher IDO expression is observed in the FAB-M5 subtype (19, 30, 31). The FAB-M5 subtype is associated with leukemia derived from a myelomonocytic lineage (38), and as aforementioned, IDO expression on mononuclear cells, including monocytes and monocyte-derived DCs has been found. As FAB-M5 is leukemia of monocytic origin and IDO can be expressed by these cells, it could account for this correlation. Contrasting this, four of the studies suggested that there was no significant difference in IDO expression between the FAB subtypes (20, 21, 25, 28). The methodologies used in these studies and those identifying a difference in IDO expression between subtypes were consistent. However, the sample size of those finding no difference were typically much larger (*n* = 650), providing more reliable statistical data. Accordingly, FAB subtypes should not be used to classify the likelihood of IDO expression.

AML mortality rates remain high despite attempts to refine treatment and develop more targeted techniques. Prognostic markers stratifying disease progression, such as the FLT3 mutation, have thus far only had limited effects on patient outcomes. Among the reviewed articles, seven studies assessed IDO expression prognosis (12, 20, 23, 25, 27, 28, 30). All associated high expression of IDO with poor prognosis, with some finding lower OS and CR attainment in IDO-positive groups. Across some of the studies, the value of IDO mRNA and Kyn levels as prognostic factors were assessed, finding that both hold potential as prognostic markers in AML (20, 25, 27, 29). Measurement of Kyn levels in patient samples are relatively easier than IDO mRNA measurements as this can be done in serum. However, a combination of both is likely to be most beneficial as a prognostic marker.

The Kyn metabolites produced by Trp cleavage can support Treg generation. The presence of Treg within the tumor environment is known to promote the development and progression of tumors by curtailing effective anti-tumor immune response, denoting poor survival in various types of cancers (39). Eight of the studies in the present review attempted to establish the role of Tregs in relation to IDO expression in AML (12, 18, 19, 24, 26, 32, 35, 36). These studies agreed that a positive correlation exists between IDO expression and Treg induction (12, 18, 19, 24, 26, 35), suggesting that in the case of AML, the immune suppressive environment may be resultant of the Treg accumulation induced by the expression of IDO. The most common CD4 Treg markers used across the studies were FoxP3 and CD25. However, they may both be transiently expressed by activated T cells in human and may not represent *bonafide* Tregs (40). CD127, is consistently expressed in high levels in activated T cells, but low or absent in Tregs, and therefore identification of CD25^+^FoxP3^+^CD127^low/−^ CD4 T cells may be more indicative of a Treg phenotype (41).

Pharmacological inhibition of IDO has been shown to break maternal immune tolerance in pregnancy, spontaneously aborting fetus, and precipitate autoimmunity in susceptible strains in mice (42, 43). Such findings provided proof-of-concept that breaking tolerance to tumor antigens in cancers where IDO is used as a route to immune escape may hold promise for cancer immunotherapy. Several studies included in this review also used the IDO inhibitor 1-MT *in vitro*. In samples from AML patients, 1-MT reduces Treg induction and Kyn levels, and increases effector T cell proliferation *in vitro* (23, 24, 26, 31, 32, 35). The 1-MT-D isoform, Indoximod, is an IDO inhibitor which exhibits a range of oral bioavailability across species, a good safety profile and the ability to slow tumor growth in preclinical studies (44, 45). In clinical trials, Indoximod has been tested as a monotherapy and in combination with chemotherapy agents where efficacy has been identified but primary endpoints have failed to be achieved, resulting in the termination of trials (45). Other IDO inhibitors that were not amongst the studies of this review, including the more potent Epacadostat, have also been trialed as therapies in many cancers but they lacked distinct objective responses (46). Preclinically, it has been suggested that a combination of IDO with checkpoint inhibitors may produce a synergistic effect that restores T cell deficits with cancer (45, 47). The ECHO 301 trial is the latest development exploring this clinically, testing Epacadostat in combination with anti-PD-1 antibody pembrolizumab in metastatic melanoma patients. This trial failed to demonstrate improved progression free survival and OS in the treatment combination compared to pembrolizumab monotherapy and thus terminated early (48).

Failure of the clinical trials to date raises questions as to where IDO inhibition could be incorporated into AML therapy, but it is important to note that although trials have taken place in other cancer types, few have focused on IDO inhibition in AML. Recently, a trial combining Indoximod with standard (7 + 3) chemotherapy was conducted in AML patients (NCT02835729) which has yet to report any findings (49). Results of this study could provide valuable insights into the rationale for continuation of trials investigating the clinical relevance of IDO inhibition therapies in AML. Nevertheless, early-mortality of AML patients with the highest IDO expression may highly likely benefit from IDO-inhibitor drugs as a component of their treatment regimen (22).

The present review is subject to limitations as only English papers could be included and a meta-analysis was not suitable due to the heterogeneity of data. It is also acknowledged that many of the papers came from the same authors and research groups/institutions therefore findings potentially could be subject to bias. The papers included in this review investigated IDO with no delineation of its isoforms or focused entirely on IDO1. It must be noted that IDO has two isoforms, IDO1 and IDO2, which exhibit high sequence homology but exhibit kinetic, functional and expression differences, with IDO2 expression limited to subsets including antigen-presenting cells (50). Current evidence suggests that IDO2 may serve an important role within immune control by supporting IDO1-dependent Treg function as part of the adaptive immune system (51). Therefore, it is important that the contribution of IDO2 in AML pathogenesis relating to Treg abundance and prognosis is explored in further studies.

To the best of our knowledge, this is the first systematic review highlighting the importance of IDO in AML progression and prognosis. IDO can be expressed in various cell types involved in AML pathogenesis and this expression can be either inducible or constitutive, differing between patients. The review identified the potential of IDO and Kyn levels as prospective prognostic markers as IDO expression correlates with worse patient outcomes. However, more robust CD4 Treg (CD4^+^CD25^+^CD127^low/−^) markers may be required to delineate the contribution of *bonafide* Treg within the tumor microenvironment where IDO activity is functionally relevant. Furthermore, the role of other similar immunosuppressive enzymes, such as Tryptophan 2,3-dioxygenase may offer novel insights into the contribution of immunomodulatory enzymatic cascade in AML pathogenesis and prognosis.

## Data Availability Statement

The raw data supporting the conclusions of this article will be made available by the authors, without undue reservation.

## Author Contributions

GW performed the search, screening and data extraction, interpreted the results, and wrote the manuscript. PK contributed to the results interpretation and writing the manuscript. LD designed the study, checked the accuracy of the data extraction, and contributed to the results interpretation and writing the manuscript. All authors contributed to the article and approved the submitted version.

## Conflict of Interest

The authors declare that the research was conducted in the absence of any commercial or financial relationships that could be construed as a potential conflict of interest.

## Abbreviations

IDO: indoleamine 2,3-dioxygenase
AML: acute myeloid leukemia
Treg: regulatory T cell
Trp: Tryptophan
Kyn: Kynurenine
FAB: French-American-British
CR: complete remission
DC: dendritic cell
OS: overall survival
1-MT: 1-methyl-tryptophan.
